# Multidimensional evaluation of offline and online education in dermatology teaching during the COVID-19 pandemic in a chinese teaching hospital: a cross-sectional study

**DOI:** 10.1186/s12909-023-04160-0

**Published:** 2023-03-29

**Authors:** Ben Wang, Mi Zhang, Zhixiang Zhao, Yingxue Huang, Ji Li, Xiang Chen, Juan Su, Mei Yi

**Affiliations:** 1 Department of Dermatology, Xiangya Hospital, Central South University, 87 Xiangya Road, Changsha, Hunan 410008 China; 2 National Clinical Research Center for Geriatric Disorders, Xiangya Hospital, Central South University, Changsha, Hunan China

**Keywords:** Multidimensional evaluation, Online education, Dermatology, COVID-19, Questionnaire

## Abstract

**Background:**

The global spread of Coronavirus disease (COVID-19) has led to the use of online teaching methods in universities, but the effect of online education on dermatology teaching remains unclear.

**Methods:**

We designed a multi-dimensional teaching evaluation form for data collection, student teaching feedback evaluation, and assessed the scores of final theoretical and clinical skill tests, to compare the effective difference between online and offline teaching of dermatology.

**Results:**

A total of 311 valid questionnaires of medical undergraduates were collected, 116 of which were enrolled for offline learning, and 195 for online learning. The average score of final theoretical test in the online teaching group had no significant difference compared with that in the offline teaching group (75.33 ± 7.37 vs.75.63 ± 7.51, *P* = 0.734). However, both scores of skin lesion recognition test and medical history collection test in the online teaching group were significantly lower than that in the offline teaching group (6.53 ± 0.86 vs. 7.10 ± 1.11, *P* < 0.001; 6.70 ± 1.16 vs. 7.62 ± 0.85, *P* < 0.001). Additionally, the scores of understanding skin lesions in the online teaching group were significantly lower than that in the offline group (*P* < 0.001), and the scores of overall understanding of skin diseases and evaluating their learning mode in the online teaching group also decreased (*P* < 0.05). Among the 195 students enrolled in the online learning group, 156 students (80.0%) recognized that the time of offline teaching should be increased.

**Conclusions:**

Both online and offline education can be used in dermatology theory teaching, but online education is less efficient in skin lesion and practical skills learning. More online teaching software with skin diseases characteristic should be developed to improve the online teaching effect.

## Background

In 2019, the Coronavirus disease (COVID-19) pandemic raged around the world, greatly changing the lives of people all over the world. To control the spread of the virus, governments around the world have taken strong control measures, such as travel restrictions, vaccinations, school closures, etc. Many schools and universities have begun to explore the model of online teaching [1]. When there is no virus transmission, offline teaching is the main traditional teaching mode. In the case of virus transmission, online teaching is the main teaching mode.

Online teaching in Chinese universities is mainly carried out through software such as Tencent conference, dinging, or Tencent QQ, where teachers and students connect through online video and voice to impart knowledge about textbooks. In the past two years, many schools, teachers and students have gradually adapted to the online teaching model, and many studies have begun to explore the advantages and disadvantages of online teaching in medical education. A cross-sectional study at the Medical School of Tongji University revealed most teachers and students were satisfied with the implementation of online education during the pandemic, but online teaching still cannot completely replace traditional offline teaching [2]. An Egyptian study said medical students reported various limitations and challenges of online medical education, and more effort is needed to improve the accessibility and structure of online medical education [3]. Researchers from various medical disciplines are also discussing the problems existing in online teaching. Some studies believe that the online teaching of rheumatology lacks emotional connections between mentors and mentees and online rheumatology education is enriched by peer review and social media activities, which are becoming major players in the time of the COVID-19 pandemic [4]. Some pediatric medical education institutions have also started to redesign the clinical learning system including a description of the learners and environment, the pedagogical principles that guided the approach, and the technological tools used in implementation [5]. More and more disciplines are exploring teaching methods and online teaching tools with disciplinary characteristics.

Different from other disciplines, dermatology is a discipline mainly based on morphology. Dermatological diseases have many specific skin lesions, and each skin lesion is different in shape, touch feeling and how it causes the patients to feel. Some studies have suggested that the online teaching of dermatology is a great challenge [6], and there are no excellent teaching tools or teaching systems. So far, only one study has shown that online dermatology education may effectively supplement traditional clinical teaching in Pediatric residents learning atopic dermatitis [7]. However, until now, there has been no specific study on the effective evaluation of online teaching in dermatology during the COVID-19 pandemic, and whether online teaching has any other impact on the dermatology study of medical students is unknown. The Dermatology Department of Xiangya Hospital has been established for more than 100 years and has been in the leading position in the teaching of dermatology in China. It has accumulated a lot of offline teaching experience and materials, but it is also constantly exploring and learning in the aspect of online teaching. Our dermatology teaching is divided into theoretical teaching, outpatient and inpatient ward probation teaching and operation teaching of clinical skills with dermatology characteristics. Due to the pandemic, all sections of dermatology teaching have been switched to online teaching.

In this study, we investigated the online and offline teaching of dermatology in several grades and majors in the past two years, evaluated the teaching effect, and conducted an online survey among students to clarify the impact of online teaching on dermatology education.

## Methods

### Participants and data collection

Undergraduate students of Xiangya Hospital who have studied dermatology and venereology during the two semesters of Year 2021 and 2022 were included, including specialties in anesthesiology, clinical medicine and stomatology. Some of these students have completed the whole course of offline teaching when there is no virus transmission, while some have completed the study of dermatology through online teaching during the virus pandemic. All the online teaching was carried out synchronously and uniformly on Tencent Conference app. All teaching handouts in our department were unified, and the online teaching content and setting were consistent. Self-designed multidimensional evaluation questionnaires were distributed to them and were to be completed online. The evaluation questionnaire contained a total of 18 self-assessment questions. Data were collected on age, gender, specialty, teaching mode, willingness to increase offline teaching time (online teaching group), 6 questions about classroom assessment (5-points scale, Cronbach’s alpha = 0.917), 4 questions about skin lesions learning assessment (5-points scale, Cronbach’s alpha = 0.856), 3 questions about overall evaluation (5-points scale, Cronbach’s alpha = 0.704). All forms are filled out in real name and with the knowledge and consent of all participants. All methods were carried out in accordance with relevant guidelines and regulations, all experimental protocols were approved by the ethnic committee of Xiangya Hospital (IRB number: 202,303,004). Informed consent was obtained from all participants.

### Teaching evaluation and assessment

After completing the undergraduate course of dermatology and venereology, all students are required to take the final theoretical examination (100 points), the clinical skill examination of medical history collection (10 points) and the skill test of skin lesion identification (10 points), making possible to evaluate the effect of the study of dermatology in multiple dimensions. This questionnaire was approved by medical educational experts in the teaching department of Xiangya Hospital. All the tests were conducted offline. According to the real name of the questionnaire, the corresponding theory and skill scores of each student can be obtained.

### Statistic analysis

All the data were imported into SPSS 24.0. For continuous variables such as age and test scores, all results are presented as means ± standard deviations, and two sample t tests or nonparametric tests were performed. Statistical value refers to the t value in two sample t tests. For categorical variables such as gender, the percentages were used, and Chi-square test was performed. All data were considered statistically significant at *P* < 0.05.

## Results

### General information

A total of 320 subjects completed the questionnaire, and 311 valid questionnaires were collected (97.2%). Among them, 116 students received offline learning, and 195 students received online learning. The average age of offline or online group was 21.78 ± 0.46 or 21.83 ± 0.52, respectively (*P* = 0.344), and there were 164 males (52.7%) and 147 females (47.3%). The data were shown in Table 1.

**Table 1:** Basic information of students in offine and online learning groups

Information	Offline learning (n = 116)	Online learning (n = 195)	Statistical value	*P* value
**Age (yr)**	21.78 ± 0.46	21.83 ± 0.52	−0.947	0.344
**Gender**			2.334	0.157
Male [n (%)]	71 (61.2)	93 (47.7)		
Female [n (%)]	45 (38.8)	102 (52.3)		
**Specialty**			3.673	0.159
Oral medicine	26 (22.4)	63 (32.3)		
Clinical Medicine	73 (62.9)	110 (56.4)		
Anesthesiology	17 (14.7)	22 (11.3)		

### Multi-dimensional teaching effect comparison of dermatological tests in offline and online learning groups

For the final theoretical examination, the average score of the online teaching group had no significant difference as compared to that of the offline teaching group (75.33 ± 7.37 vs.75.63 ± 7.51, *P* = 0.734). For tests related to clinical skills, we observed a very significant difference. The score of skin lesion recognition test in the online teaching group was significantly lower than that of offline teaching group (6.53 ± 0.86 vs. 7.10 ± 1.11, Cohen’d value = 0.574, *P* < 0.001), and the score of medical history collection test in the online teaching group was also significantly lower than that of offline teaching group (6.70 ± 1.16 vs. 7.62 ± 0.85, Cohen’d value = 0.905, *P* < 0.001). The data were shown in Table 2.

**Table 2:** Multi-dimensional teaching effect comparison of dermatological tests in offine and online teaching groups

Test outcomes	Offline teaching (n = 116)	Online teaching (n = 195)	Statistical value^#^	*P* value	Cohen’d value
Final theoretical test scores (100 points)	75.63 ± 7.51	75.33 ± 7.37	0.340	0.734	0.040
Skin lesion recognition test scores (10 points)	7.10 ± 1.11	6.53 ± 0.86	5.060	**< 0.001**	0.574
Medical history collection test (10 points)	7.62 ± 0.85	6.70 ± 1.16	7.442	**< 0.001**	0.905

# Statistical value refers to the t value obtained in the t test

### Multi-dimensional comparison of student assessment in offline and online learning groups

To evaluate the feedback effect of students in the online teaching, we developed a multi-dimensional evaluation index according to the corresponding teaching methods and combined with the teaching characteristics of dermatology, and the results were shown in Table 3. For the classroom assessment, the self-assessment scores of the concentration level of classroom learning, interest in dermatology learning, the interaction with the teacher and classmates in the online teaching group were significantly lower than that in the offline group (4.51 ± 0.59 vs. 4.76 ± 0.50, 4.75 ± 0.46 vs. 4.88 ± 0.40, 4.49 ± 0.66 vs. 4.66 ± 0.58, 4.37 ± 0.79 vs. 4.66 ± 0.61, *P* < 0.05), respectively, suggesting students’ overall evaluation of online teaching class is relatively low. However, the score of improved independent learning ability in the online teaching group was significantly higher than that in the offline group (4.66 ± 0.55 vs. 4.47 ± 0.73, *P* = 0.01), and there was no difference between the two groups in the learning enthusiasm (*P* = 0.064). For the skin lesions learning assessment, two index of the online teaching group (understanding skin lesions (touching skin lesions) and the patients’ perception) were significantly lower than that of the offline teaching group (3.50 ± 1.11 vs. 4.55 ± 0.77, 4.18 ± 0.85 vs. 4.55 ± 0.80, Cohen’d value = 1.100 & 0.448, *P* < 0.001), while other two scores of the online teaching group (understanding skin lesions (identify skin lesions) and skin lesions (the scope and severity of skin lesions)) had no significant differences (*P* > 0.05). Finally, there are also some differences in students’ overall evaluation of dermatology teaching. The score of overall understanding of skin diseases in the online teaching group was lower than that in the offline group (4.31 ± 0.73 vs. 4.49 ± 0.75, *P* = 0.04); and the score of evaluating their own learning mode is also significantly lower in the online teaching group (4.13 ± 0.57 vs. 4.50 ± 0.60, Cohen’d value = 0.632, *P* < 0.001), but there was no significant difference in overall teaching satisfaction (*P* = 0.254). Among the 195 online learning students, 156 students (80.0%) believed that the time of offline teaching should be increased.

**Table 3:** Multi-dimensional comparison of student assessment in offine and online teaching groups

Items	Offline learning (n = 116)	Online learning (n = 195)	Statistical value^#^	*P* value	Cohen’d value
**Classroom assessment** (5-points scale)					
The concentration level of classroom learning	4.76 ± 0.50	4.51 ± 0.59	3.762	**< 0.001**	0.475
Interest in dermatology learning	4.88 ± 0.40	4.75 ± 0.46	2.459	**0.014**	0.302
The interaction with the teacher	4.66 ± 0.58	4.49 ± 0.66	2.272	**0.024**	0.274
The interaction with classmates	4.66 ± 0.61	4.37 ± 0.79	3.372	**0.001**	0.411
Learning enthusiasm	4.69 ± 0.55	4.57 ± 0.56	1.856	0.064	0.216
Improved independent learning ability	4.47 ± 0.73	4.66 ± 0.55	−2.58	**0.01**	0.294
**Skin lesions learning assessment** (5-points scale)					
The degree of understanding skin lesions (identify skin lesions)	4.60 ± 0.70	4.47 ± 0.66	1.731	0.084	0.191
The degree of understanding skin lesions (touch skin lesions)	4.55 ± 0.77	3.50 ± 1.11	9.000	**< 0.001**	1.100
The degree of understanding skin lesions (the scope and severity of skin lesions)	4.53 ± 0.75	4.39 ± 0.77	1.524	0.129	0.184
Understand the patients’ perception (such as itching, tingling, etc.)	4.55 ± 0.80	4.18 ± 0.85	3.779	**< 0.001**	0.448
**Overall evaluation** (5-points scale)					
Overall understanding of skin diseases	4.49 ± 0.75	4.31 ± 0.73	2.060	**0.04**	0.243
Comments on your learning mode	4.50 ± 0.60	4.13 ± 0.57	5.445	**< 0.001**	0.632
Satisfactory value	4.74 ± 0.55	4.67 ± 0.50	1.143	0.254	0.133

# Statistical value refers to the t value obtained in the t test

## Discussion

The novel COVID-19 outbreak has led to the emergence of online teaching in many universities around the world for more than two years [8]. The online teaching system is constantly supplemented and improved, and more and more research on online teaching is conducted to provide more suggestions on online teaching [9, 10]. Our research provides more data and theoretical basis for online teaching in the field of dermatology teaching.

As young college teachers, we have a lot of confusions in the process of online dermatology teaching. Most internships and clinical skills practice have been transferred to online teaching, so students cannot accurately experience the learning of skin lesions and the patients’ perception. Meanwhile, the medical history collection can only be completed between students, and they cannot deeply understand the feelings of patients themselves and various emergencies in the process of medical history collection, which results in the limitations of online learning of clinical practice ability [11, 12]. Our research results also reflect this teaching problem. Many students can learn from textbooks on their own, do well in the theoretical study of dermatology, and get satisfactory results in the final examination, but the clinical practice of identifying skin lesions cannot be the same as the theoretical learning process. We selected some confused outpatient clinical pictures for students to judge and write the main points of skin disease inquiry, and the scores were not good. The reason is that the profound understanding of morphology cannot be completely learned from the books [13]. It is necessary to have face-to-face communication with patients through outpatient and ward internship and practice, and students can well define skin diseases combined with comprehensive actual touching of skin lesions, some dermatological physical examination (such as detection of Nikolsky sign, dermographism and Auspitz sign), actual feelings of patients and other clinical data [14–16].

Furthermore, from the learning feedback and questionnaire survey of students, it also reflected the problems of clinical skills test. The students in the online teaching group reported less interaction with their classmates and teachers, some limitations in palpation and understanding of skin lesions, and some limitations in their overall knowledge of skin diseases. These findings are in line with our expectations and are consistent with other studies on online teaching [17, 18], which has pointed out many similar limitations of online teaching in other disciplines. It is easy to understand that there are space limitations in online teaching, and students’ focus will be scattered, and they will not be able to communicate with patients. This poses a challenge to the teaching of morphology. We are also thinking about whether we can develop better online teaching software or system through artificial intelligence technology. Through haptic simulation system and Virtual Reality system [19, 20], students can feel the touching feeling of skin lesions online or conduct physical examination of skin diseases, so that students can better experience skin lesions in their own position. It may be better to improve the effect of online teaching in dermatology.

### Limitations

Firstly, this study was only conducted in our hospital, and perhaps the online teaching system in our hospital is not perfect enough, which may also lead to insufficient teaching effect. Therefore, a multi-center study with a larger sample size is needed to comprehensively evaluate the shortcomings of online teaching in dermatology. Secondly, our survey was conducted through an online questionnaire, and the validity of the questionnaire has not been verified, and recall bias and misclassification may exist. Thirdly, we think that there is a lack of more evaluation methods in the examination of clinical skills. If necessary, we can add the actual operation such as fungal microscopic examination and Nikolsky sign examination to evaluate.

## Conclusions

Collectively, both online and offline education can be used in dermatology theory teaching. However, despite the advantages of online teaching, online education is difficult to completely replace traditional offline teaching in skin lesion and practical skills learning. Some new artificial intelligence (AI) technologies could be applied, such as haptic simulation system could better let students experience the touch of skin lesions, or Virtual Reality system could simulate the three-dimensional structure of skin more realistically or allow students to be in a specific medical scene more realistically. Therefore, to improve the teaching effect, we need to develop more online teaching software with skin diseases characteristic combined with new AI technologies.

## Data Availability

The datasets used and/or analysed during the current study are available from the corresponding author on reasonable request.
